# Integrated analysis of circRNA, lncRNA, miRNA and mRNA to reveal the ceRNA regulatory network of postnatal skeletal muscle development in Ningxiang pig

**DOI:** 10.3389/fcell.2023.1185823

**Published:** 2023-07-03

**Authors:** Zonggang Yu, Xueli Xu, Nini Ai, Kaiming Wang, Peiwen Zhang, Xintong Li, Sui LiuFu, Xiaolin Liu, Jun Jiang, Jingjing Gu, Ning Gao, Haiming Ma

**Affiliations:** ^1^ College of Animal Science and Technology, Hunan Agricultural University, Changsha, China; ^2^ Guangdong Laboratory for Lingnan Modern Agriculture, Guangzhou, China

**Keywords:** ceRNA network, circRNA, lncRNA, Ningxiang pig, skeletal muscle development, whole transcriptome

## Abstract

**Introduction:** The development of skeletal muscle is regulated by regulatory factors of genes and non-coding RNAs (ncRNAs).

**Methods:** The objective of this study was to understand the transformation of muscle fiber type in the longissimus dorsi muscle of male Ningxiang pigs at four different growth stages (30, 90, 150, and 210 days after birth, n = 3) by histological analysis and whole transcriptome sequencing. Additionally, the study investigated the expression patterns of various RNAs involved in muscle fiber transformation and constructed a regulatory network for competing endogenous RNA (ceRNA) that includes circular RNA (circRNA)/long non-coding RNA (lncRNA)-microRNA (miRNA)-messenger RNA (mRNA).

**Results:** Histomorphology analysis showed that the diameter of muscle fiber reached its maximum at 150 days after birth. The slow muscle fiber transformation showed a pattern of initial decrease followed by an increase. 29,963 circRNAs, 2,683 lncRNAs, 986 miRNAs and 22,411 mRNAs with expression level ≥0 were identified by whole transcriptome sequencing. Furthermore, 642 differentially expressed circRNAs (DEc), 505 differentially expressed lncRNAs (DEl), 316 differentially expressed miRNAs (DEmi) and 6,090 differentially expressed mRNAs (DEm) were identified by differential expression analysis. Functions of differentially expressed mRNA were identified by gene ontology (GO) and Kyoto Encyclopedia of Genes and Genomes (KEGG). GO enrichment analysis indicates that 40 known genes and 6 new genes are associated with skeletal muscle development. Additionally, KEGG analysis shows that these genes regulate skeletal muscle development via MAPK, FoxO, Hedgehog, PI3K-Akt, Notch, VEGF and other signaling pathways. Through protein-protein interaction (PPI) and transcription factor prediction (TFP), the action mode of skeletal muscle-related genes was explored. PPI analysis showed that there were stable interactions among 19 proteins, meanwhile, TFP analysis predicted 22 transcription factors such as HMG20B, MYF6, MYOD1 and MYOG, and 12 of the 19 interacting proteins were transcription factors. The regulatory network of ceRNA related to skeletal muscle development was constructed based on the correlation of various RNA expression levels and the targeted binding characteristics with miRNA. The regulatory network included 31 DEms, 59 miRNAs, 667 circRNAs and 224 lncRNAs.

**conclusion:** Overall, the study revealed the role of ceRNA regulatory network in the transformation of skeletal muscle fiber types in Ningxiang pigs, which contributes to the understanding of ceRNA regulatory network in Ningxiang pigs during the skeletal muscle development period.

## 1 Introduction

The Ningxiang pig, a Chinese native pig known for its high-fat content, has the advantages of tolerance to rough feeding, a high reproduction rate, a mild temperament, and strong adaptability (Feng et al., 2012). Besides, the Ningxiang pig has a unique flavor due to its high intramuscular fat content. For instance, the content of arachidonic acid and 22 carbon hexaenoic acid is significantly higher than those of lean pig breeds (He et al., 2012). Previous studies mainly focused on the characteristics of Ningxiang pig germplasm, genetic composition (Gao et al., 2022), and molecular mechanism of fat deposition (Li B. et al., 2021; Gong et al., 2021; Li et al., 2022). The molecular mechanism of skeletal muscle development in Ningxiang pig is rarely studied. Therefore, it is of great interest to explore the molecular mechanism of skeletal muscle development in Ningxiang pigs, which contributes to further understand the fat deposition in skeletal muscle.

Pig is not only one of the major meat animals, but also used as a major mammal model to study the pathological changes including organ transplantation, digestive diseases, blood dynamics, and diabetes (Verma, Rettenmeier, and Schmitz-Spanke, 2011). Studying the underlying mechanisms of skeletal muscle development is not only beneficial to improve meat quality by genetic modification, but also may provide a new perspective for the study of human muscle development and muscle-related diseases. Skeletal muscle is a post-mitotic tissue composed of multinuclear muscle fibers, derived from the division of monomyocytes (Yin, Price, and Rudnicki, 2013). Skeletal muscle development in pigs is characterized by hyperplasia and hypertrophy. Hyperplasia occurs mainly during the embryonic period and is manifested by an increase in the number of muscle fibers. Hypertrophy mainly occurs after birth, mainly manifested by the increase of muscle fiber volume and the transformation of muscle fiber types (Ashmore, Addis, and Doerr, 1973; Suzuki and Cassens, 1980). The primary and secondary myotubes of skeletal muscle in pigs are mainly formed at the age of 33 days and 65 days and the number of muscle fiber reaches to the plateau mainly at the 85–95 days of pregnancy (Wigmore and Stickland, 1983; Zhao Y. et al., 2015). The research on the development profiles of pig skeletal muscle mainly focuses on different embryonic stages (Hong et al., 2019), postnatal muscle fiber types (Li et al., 2020; Wang et al., 2022a; Tan et al., 2023) and breeds (Qi et al., 2022; Zhuang et al., 2022). There are few reports on the development of pig skeletal muscle at different stages after birth. Zhuang et al. explored the molecular mechanism of muscle development in Lantang and Landrace pigs 1 and 90 days after birth by high-throughput sequencing (Zhuang et al., 2022). Li et al. reported the expression of circRNA in skeletal muscle of Mashen pig and Yorkshire pig in three stages after birth, and explored the molecular mechanism of muscle development (Li M. et al., 2021). Despite these progresses, the dynamic expression of RNA in porcine skeletal muscle development after birth is still unclear, and skeletal muscle development needs further systematic exploration.

RNA sequencing is a key technology to explore gene function and deeply study molecular mechanism. A large number of miRNA, lncRNA, circRNA and mRNA can be obtained by RNA sequencing. Gene is a key factor affecting the development process, gene expression is regulated not only by transcription factors, but also by ncRNA. NcRNA refers to the nucleic acid sequence without coding ability, including miRNA, long-chain noncoding RNA (lncRNA) and circRNA. LncRNA and circRNA can regulate the expression of mRNA and affect the biological process by competitively binding to miRNA (Cesana et al., 2011; Salmena et al., 2011; Hansen et al., 2013). Li et al. found that CDR1as could induce the differentiation of porcine myoblasts by adsorbing miR-7 and up-regulating the expression of IGF1R (Li et al., 2019). By sequencing the longissimus dorsi muscle of Jinfen pig, Sun et al. discovered that circCSDE1 regulated the expression of CDK16 by binding to miR-21-3p, and verified on C2C12 that circCSDE1 promoted myoblast proliferation and inhibited myoblast differentiation (Sun et al., 2022). Li et al. examined that circIGF1R promoted the differentiation of porcine myoblasts by adsorbing miR-16 regulatory gene expression (Li et al., 2023). Wang et al. reported that Gm10561 can upregulate E2F3 and MEF2C via targeted binding to miR-432 and promote the proliferation and differentiation of muscle cells in pigs and mice (Wang et al., 2022b). Tan et al. constructed the regulatory network of G1430 (lncRNA)-miR-133/SRF/GosB regulating muscle growth and development by sequencing and bioinformatics analysis of the longissimus dorsi muscle of Qingyu pigs at three postnatal periods (Tan et al., 2021). Duo et al. found that MyHC IIA/X-AS adsorbed miR-130 upregulated MyHC IIX and promoted the transformation of porcine myoblasts from fast muscle fibers to slow muscle fibers (Dou et al., 2020). However, the regulatory network of ceRNA mediated by circNRA/lncRNA is rarely reported in the skeletal muscle development of Ningxiang pigs.

The objective of the study is to explore the key role of ceRNA regulatory network in the skeletal muscle development of Ningxiang pigs. The longissimus dorsi muscle of Ningxiang pigs aged 30 (Weaned piglets), 90 (nursing pigs), 150 (early fattening pigs) and 210 (late fattening pigs) days after birth was collected for histological morphological analysis and transcriptome sequencing. The key genes related to skeletal muscle and the key circRNA and lncRNA regulating the expression of genes were obtained by bioinformatics analysis. Finally, the regulatory network of ceRNA mediated by circRNA/lncRNA was constructed, which provides reference for the further study of skeletal muscle development.

## 2 Materials and methods

### 2.1 Ethics statement

The experimental animal procedures were followed in accordance with the approved protocols of Animal Science and Technology College of Hunan Agriculture University (No. 2021–13).

### 2.2 Experimental design and samples preparation

Twelve healthy male Ningxiang pigs were selected, including three Ningxiang pigs at 30, 90, 150 and 210 days after birth. The pigs at the same stage were full-sibs and half-sibs at different stages. The experimental pigs came from the pig farm of Ningxiang Dalong Animal Husbandry Technology Co., LTD. All pigs in the study were provided *ad libitum* access to water and feed. *Longissimus dorsi* (LD) muscle of the fourth to last ribs was collected within 30 min after slaughter. The muscle samples were frozen in liquid nitrogen and then transferred to −80°C for storage until RNA extraction.

### 2.3 Muscle tissue immunofluorescence staining

The LD muscle samples from pigs at four stages stored in 4% paraformaldehyde were embedded in paraffin. LD muscles were sliced into 10 μm thickness sections by a pathology microtome (Leica, China). Sections were blocked with 3% bovine serum albumin (BSA) for 30 min and then incubated with primary antibodies (slow MyHC, 1:300, Aifangbio, Cat. No. AF30106; fast MyHC, 1:400, Sevicebio, Cat. No. GB112130). After being washed with PBS (pH 7.4), muscle sections were incubated with goat anti-rabbit IgG (H + L)-Cy3 (1:200, Aifangbio, Cat. No. AFSA006) or goat anti-rabbit IgG (H + L)-Alexa Fluor (1:100, Aifangbio, Cat. No. AFSA005). DAPI (Solarbio, Cat. No. C0060) was used to stain the nucleus. Images were collected by Fluorescent Microscopy (Nikon, Japan). The numbers of slow-twitch fiber and fast-twitch fiber were counted by Image-Pro Plus 6.0.

### 2.4 RNA isolation, library construction and sequencing

Total RNA was extracted from longissimus dorsi muscle of Ningxiang pigs by Trizol reagent (Invitrogen, Life Technologies, Carlsbad, CA, United States) following the manufacturer’s procedure. The amount and purity of the extracted RNA were detected by Nanodrop 2000 (NanoDrop Technologies, Wilmington, DE, United States), RNA integrity was detected by agarose gel electrophoresis and RIN value was determined by Agilent 2100 (Agilent Technologies, Santa Clara, CA, United States). A single library construction requires a total RNA of 5 μg, concentration ≥250 ng/μL, OD260/280 between 1.8 and 2.2. Ribosomal RNA was removed using the Ribo-Zero Magnetic kit (Epicentre, Madison, WI, United States). Next, TruSeqTM Stranded Total RNA Library Prep Kit is used to construct a strand specific library to detect circRNA, lncRNA and mRNA. A small RNA library was constructed using Illumina TruSeq Small RNA kit to detect miRNA. The strand specific library and small RNA library were sequenced on HiSeq 4000 platform by PE150 and SE50 respectively. Deep sequencing was performed by Shanghai Majorbio Bio-pharm Technology Co., Ltd. (Shanghai, China).

### 2.5 RNA-seq data analysis

To obtain clean data, raw data was verifed by Fastp v0.23.3 (https://github.com/OpenGene/fastp), then raw paired end reads were trimmed and quality controlled by SeqPrep v1.2 (https://github.com/jstjohn/SeqPrep) and Sickle v1.33 (https://github.com/najoshi/sickle) with default parameters. Hisat2 v2.2.1 (https://ccb.jhu.edu/software/hisat2/index.shtml) software was used to map the reads to Ningxiang pig reference genome (accession number: PRJNA531381) to obtain known genes with default parameter (Kim, Langmead, and Salzberg, 2015). The novel transcript is identified by Stringtie v2.2.0 (https://ccb.jhu.edu/software/stringtie). The known lncRNA is obtained from the sum of NONCODE v6.0, Ensembl 2022, NCBI, UCSC, LncRNAdb v2.0, GENCODE 2021, LncRNA Disease and other databases. The procedures for screening candidate lncRNA are as follows: 1) remove mRNA (transcripts and their splices) from the genome database; 2) use gffcompare information to screen intergenic lncRNA, intronic lncRNA, anti-sense lncRNA three different types of lncRNA;3) select transcripts with length ≥200bp, Exon ≥2, ORFs ≤300bp, count ≥3. The novel lncRNA was identified by protein domain analysis of CPC (Kang et al., 2017), CNCI (Sun et al., 2013) and Pfam v35 (Sonnhammer et al., 1998), and the parameters were as follows: CPC score <0, cnci score <0, Pfam not significat. CircRNA was identified by CIRI software v1.2.2 (Gao, Zhang, and Zhao, 2018). The miRBase database (miRBase 21, http://www.mirbase.org/) was used to obtain known miRNA. SRNA which is not included in Rfam and miRBase is mapped to the reference genome and the secondary structure is predicted by miRDeep2 v0.1.3 (https://www.mdc-berlin.de/content/mirdeep2) (Mackowiak, 2011). According to the prediction results, the novel miRNA is identified by filtering with the characteristics of Dicer restriction site information and energy value. The expression levels of mRNA, miRNA and lncRNA are based on TPM (Zhou et al., 2010), while the expression levels of circRNA are based on RPM. Differential expression analysis was performed by DEGseq2 v0.2.0 (Love, Huber, and Anders, 2014). Significantly differentially expressed circRNA selection criteria for: *p* < 0.05 and | log2FC | ≥2.

### 2.6 Functional enrichment analysis

Gene Ontology (GO) and Kyoto Encyclopedia of Genes and Genomes (KEGG) enrichment analysis was performed for differentially expressed (DE) mRNA. GO enrichment analysis was performed by Goatools (https://github.com/tanghaibao/Goatools). KOBAS v3.0 (http://kobas.cbi.pku.edu.cn) was used for KEGG pathway enrichment analysis (Xie et al., 2011). GO terms and KEGG pathways calibration *p* values (corrected *p*-value) ≤ 0.05 is considered as significant enrichment. Transcription factor detection was completed using AnimalTFDB 3.0 (Hu et al., 2019). Protein-protein interaction (PPI) networks were predicted by STRING database v11.5 (https://string-db.org/) using genes of skeletal muscle-related in human, pig and mouse species and interactomes were analyzed with the Network Analyser plugin of Cytoscape v3.9.1 (https://cytoscape.org/) using the most stringent criteria (Smoot et al., 2011).

### 2.7 Construction of the CircRNA/lncRNA-miRNA-mRNA network

Since circRNA can be used as a molecular sponge for miRNA to regulate target gene expression (Hansen et al., 2013). CeRNA regulatory network is implemented by Majorbio Cloud Platform (https://cloud.majorbio.com/). MiRanda software (http://www.miranda.org/) was used to predict the binding sites of miRNA with lncRNA, circRNA and mRNA, including 981 miRNAs, 12,474 mRNAs, 2,004 lncRNAs, circRNAs. Relationship pairs in the network showed a negative correlation between miRNA and ceRNA expression and a positive correlation between ceRNA and ceRNA expression, with a significant level of *p* < 0.05. lncRNA (circRNA)-miRNA-mRNA correlation network was constructed by binding sites prediction by miRanda software and expression correlation. GO annotation analysis was performed on differentially expressed mRNA. Finally, we selected mRNAs enriched in GO terms related to skeletal muscle and constructed circRNA/lncRNA-miRNA-mRNA interaction network. The constructed interactive network is displayed visually with cytoscape software.

### 2.8 Verification of DE-RNA and cyclization of circRNA

Six DE circRNAs, four DE mRNAs and nine DE lncRNAs in skeletal muscle-related interaction networks were selected randomly for real-time quantitative PCR verification. According to the previous research, two circRNAs in the network were verified by divergent primers and convergent primers (Memczak et al., 2013a; Jeck et al., 2013). Primers designed “face to face” and “back to back” according to sequences containing splicing sites are convergent and divergent primers, respectively. Convergent primers have amplification products in both cDNA and gDNA, whereas divergent primers have amplification products only in cDNA. Comparisons were made to illustrate that circRNA are formed by back-splicing. The convergent primer and divergent primer sequences are shown in Supplementary Table S1. The PCR products of cDNA and genomic DNA amplified by convergent primers and divergent primers were analyzed by agarose gel electrophoresis. The back-splicing site of circRNA was verified by Sanger sequencing. Trizol reagent was used to isolate tissue RNA. RevertAid First Strand cDNA Synthesis with DNase I Kit (Thermo fisher, usa) was used for reverse transcription and Synthesis of cDNA according to the instructions. Hieff^®^ QPCR SYBR Green Master Mix (No Rox) (Yeasen, Shanghai, china) was used to detect gene expression. The relative expression of RNA was calculated by 2^−ΔΔCT^ method. Glyceraldehyde-3-phosphate dehydrogenase (GAPDH) was used as an internal reference gene. Primers were designed using Primer 5.0 software. Primer sequences synthesis and Sanger sequencing were completed by Beijing tsingke Biotechnology Co., Ltd. Three independent replicates were used for each assay, and results are presented as means and standard error of the mean (SEM).

### 2.9 Statistical analysis

The data of RT-qPCR were analyzed by paired *t*-test using SPSS 21.0 software. Origin 2021 is then used for graph visualization. A *p* < 0.05 was considered a significant difference.

## 3 Results

### 3.1 Detection of skeletal muscle fiber types in Ningxiang pigs in different periods

**FIGURE 1:**
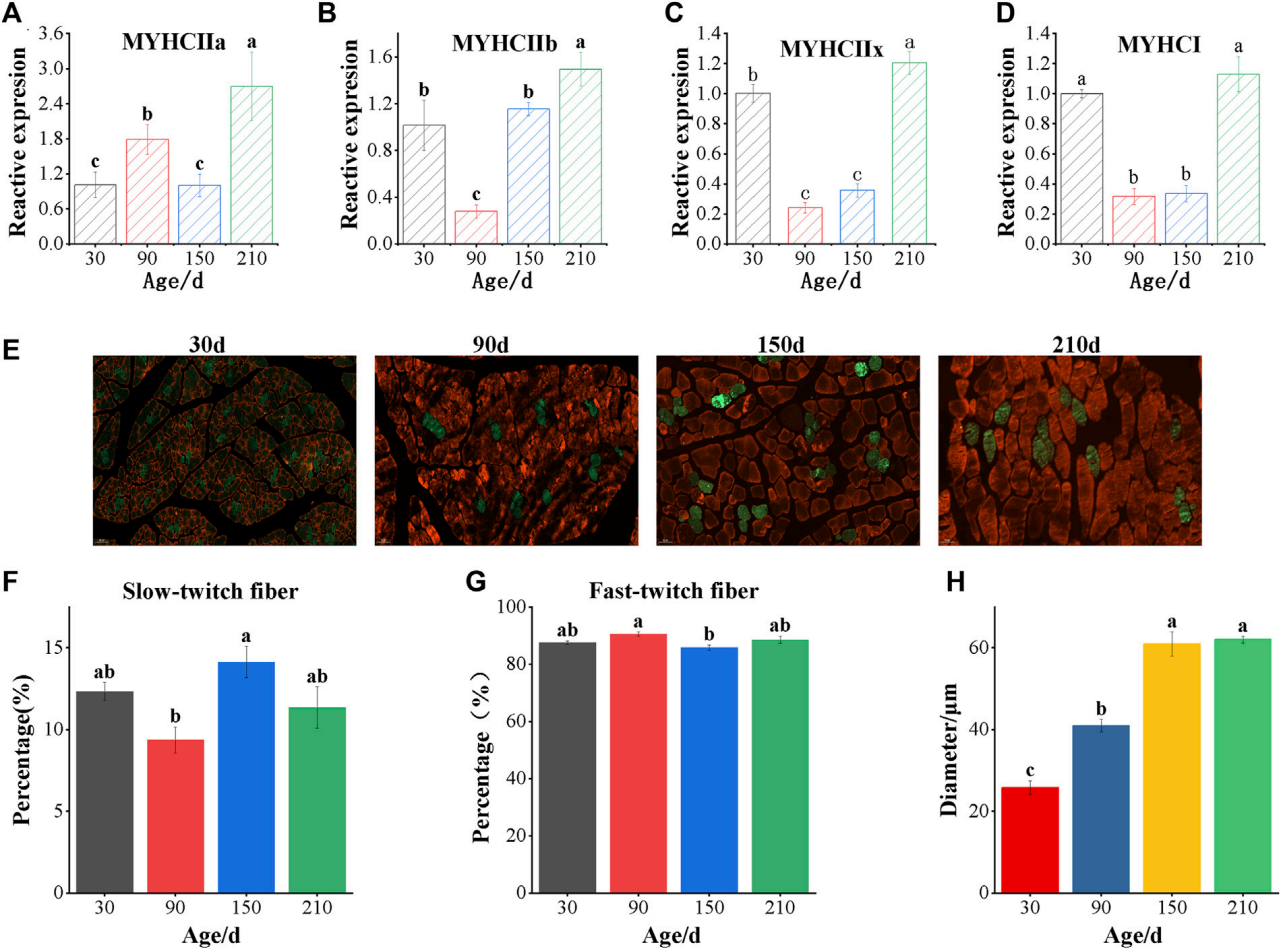
Detection of muscle fiber types of longissimus dorsi in Ningxiang pigs of different ages. **(A–D)** Real-time quantitative PCR analyzed the mRNA levels of *MYHC IIa, MYHC IIb, MYHC IIx* and *MYHC* *I* **(E)** The immunofluorescence staining of Longissimus dorsi muscle. The white scale bars represent 50 μm. Red indicates fast-twitch fibers and green indicates slow-twitch fibers. Immunofluorescence analyzed the percentage of slow-twitch fiber **(F)**, fast-twitch fiber **(G)** and muscle fiber diameter **(H)**. Data are presented as the mean ± SEM. n = 3. ^a, b, c^ Bar with different letter indicates significant difference (*p* < 0.05).

As can be seen from Figure 1, the muscle fiber diameter increases with age, but no longer increases when it grows to 150 days (Figures 1E,H). The proportion of slow muscle fibers was significantly lower at 90 days after birth than that at other ages, and reached the highest at 150 days after birth (Figures 1F,G). The results of mRNA expression analysis of muscle fiber marker gene showed that the expression of myosin heavy chain I (*MYHCI*) and myosin heavy chain IIx (*MYHCIIx*) decreased at first and then increased with the increase of age (Figures 1C,D). The expression of myosin heavy chain IIa (*MYHCIIa*) at 210 days was significantly higher than that in the other three stages (*p* < 0.05), and that at 90 days was significantly higher than that at 30 and 150 days (*p* < 0.05) (Figure 1A). The expression of myosin heavy chain IIb (*MYHCIIb*) at 210 days was significantly higher than that at other ages (*p* < 0.05), showing a trend of first decreasing to the lowest and then increasing to the highest (Figure 1B).

### 3.2 Quality control of raw data and identification of four types of RNA

To understand the expression profiles of ncRNA and mRNA in four different growth stages of Ningxiang pig, ribosomal removal RNA sequencing and small RNA sequencing were carried out. Firstly, 12 ribosome-deleted RNA chain specific libraries and 12 small RNA libraries of longissimus dorsi muscle at four stages were constructed, which were named NX30 dM (1–3), NX90 dM (1–3), NX150 dM (1–3) and NX210 dM (1–3) respectively. Then we carried out RNA-seq on the HiSeq 4000 platform and obtained about 1356.79M raw reads from 12 strand-specific libraries. After quality control, 1327.70M clean reads were obtained, and the Q30 value was 95.47%. The clean data of 1155.90 Mb was mapped with the genome of Ningxiang pig (Supplementary Tables S2-1). The clean reads are mainly distributed in the CDS region (about 74.41%) (Supplementary Figure S1A and Supplementary Table S3-2), and the chromosome distribution analysis shows that it is mainly distributed on chromosomes 15, 12, 1 and 14 (Supplementary Figure S1B and Supplementary Table S3-1). A total of 221,562,283 raw reads were obtained from 12 small RNA libraries, and 173,915,270 clean reads were obtained after quality control, with a Q30 value of 96.77% and 145,629,431 useful reads, with an average mapping rate of 91.77% (Supplementary Table S2-2).

**FIGURE 2:**
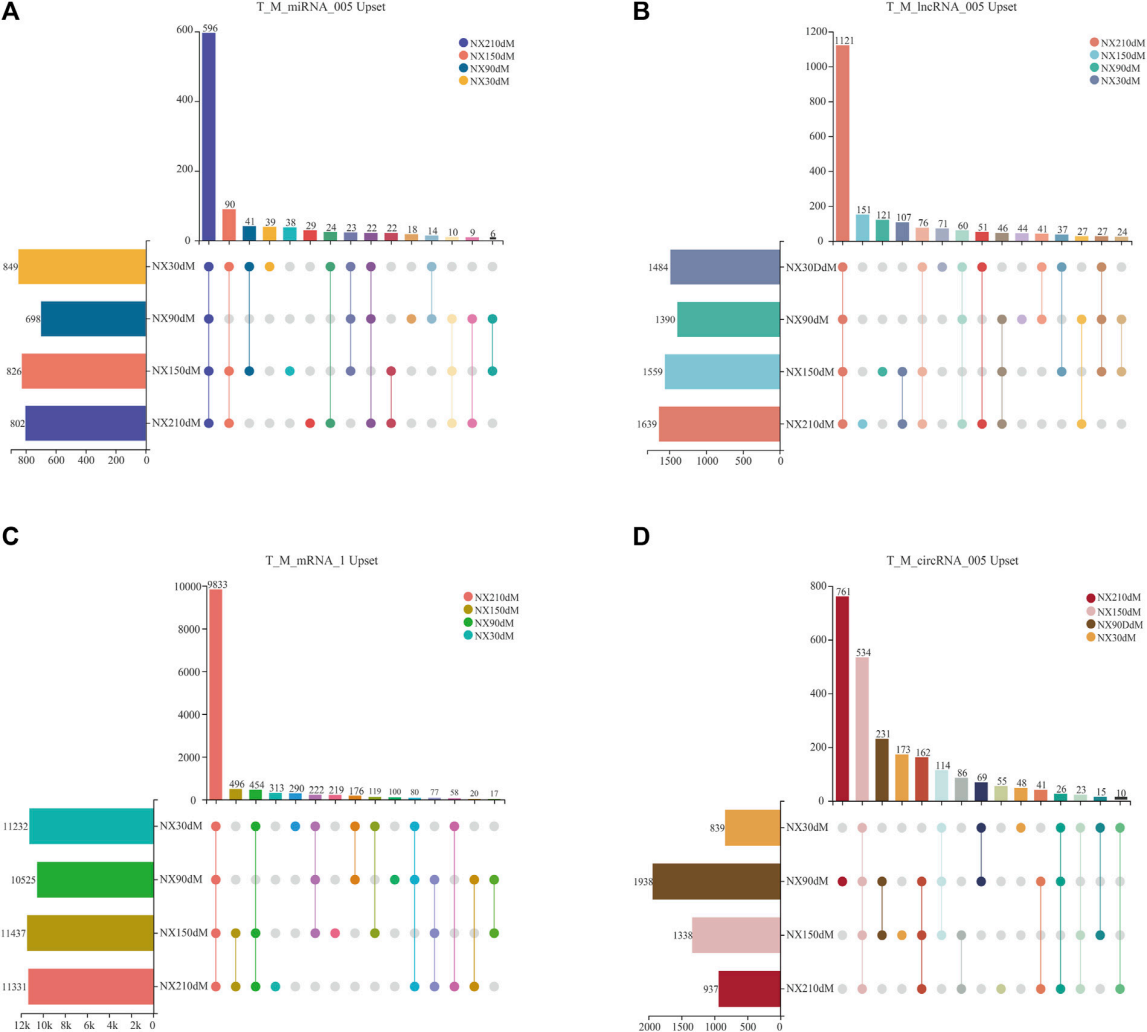
Upset analysis of four types of RNA by expression level screening. **(A–B)** upset of lncRNAs and miRNAs with TPM>0.05. **(C)** upset of mRNAs screened with TMP>1. **(D)** upset of circRNAs screened with RPM>0.05. Solid colored dots in the figure indicate presence in a stage. The number at the top of the bar represents the number of transcripts.

All kinds of RNA were refined by expression level (Venø et al., 2015) and only ncRNA with expression level >0.05 was retained, including 2,004 lncRNAs, 2,348 circRNAs, 981 miRNAs and 12,474 mRNAs with expression level >1 (Figures 2A–D and Supplementary Table S4-(1–4)).

These 981 miRNAs were 849, 698, 826 and 802 in each stage, including 596 miRNAs in four stages, with 39, 18, 38 and 29 unique miRNAs in each stage (Figure 2A). 1,484, 1,390, 1,558 and 1,639 lncRNAs of Ningxiang pig skeletal muscle were obtained in 30d, 90d, 150 d and 210d, respectively, of which there were 1,121 lncRNAs in four stages and 71, 44, 121, 151 specific lncRNAs in each stage (Figure 2B). There are 12,474 mRNAs transcripts with TPM>1 in four stages of muscle, including 11, 232, 10, 525, 11, 437 and 11, 331 at 30, 90, 150 and 210 days, respectively. There are 290, 100, 219 and 313 unique mRNAs in each stage and there are 9,833 mRNAs in four stages (Figure 2C). The sum of 2,348 circRNAs distributed in each stage was 938, 1,938, 1,338 and 937, respectively, of which there were 534 circRNAs in four stages and 48, 761, 173, 55 specific circRNAs in each stage (Figure 2D). Cluster analysis of RNA is shown in Supplementary Figure S2-(A-D).

### 3.3 Differential expression analysis of all kinds of RNA in skeletal muscle development of Ningxiang pigs at different stages

**FIGURE 3:**
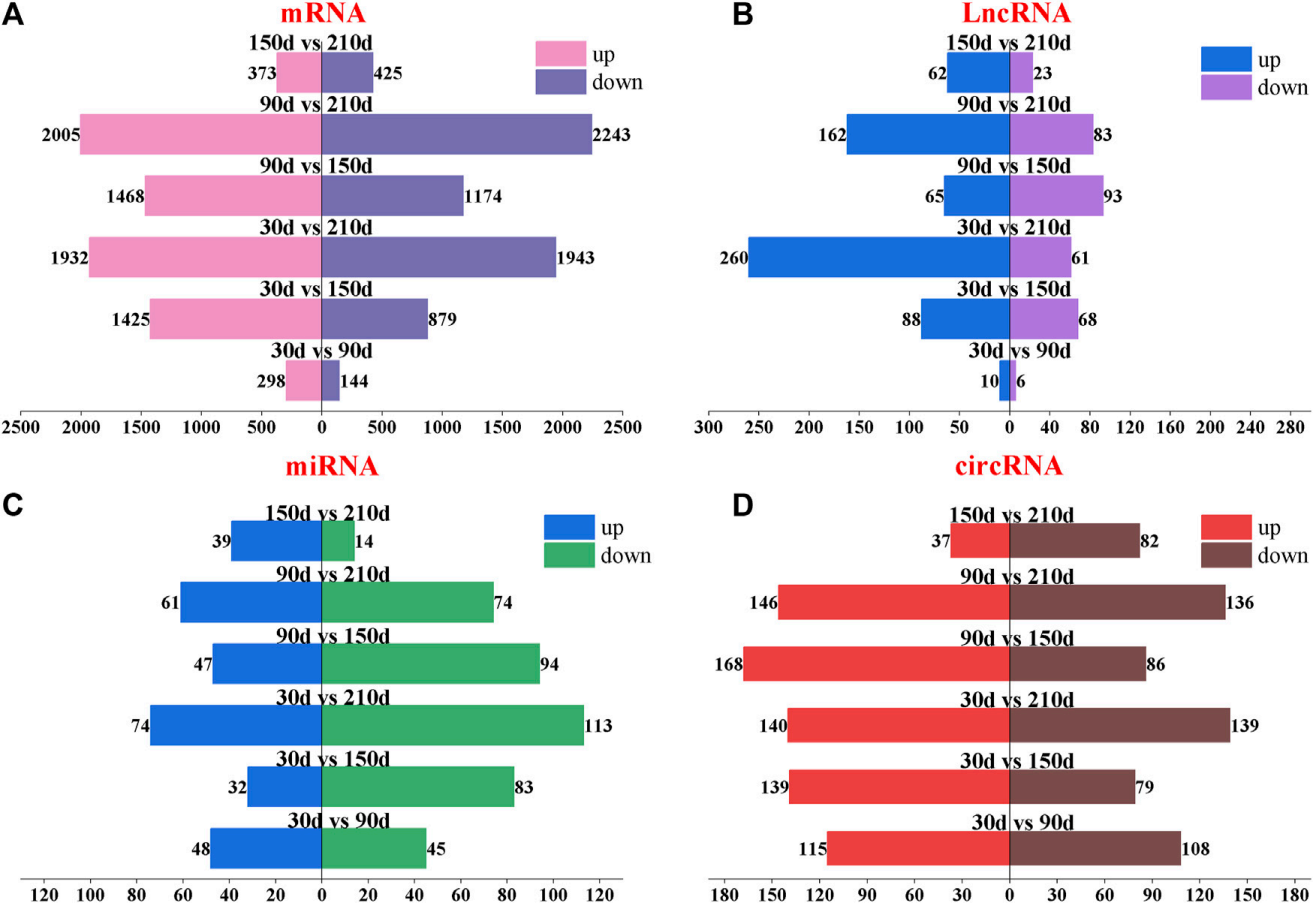
Paired difference analysis of four types of transcripts of Longissimus dorsi muscle of Ningxiang pigs at four postnatal stages (30d, 90d, 150d and 210 d). **(A–D)** mRNA, lncRNA, miRNA and circRNA, respectively. The number at the top of the bar chart indicates the number of upregulated or downregulated transcripts. The difference groups are above the center of the bar chart.

To further understand the expression changes of the whole transcript, the differential expression analysis of ncRNA and mRNA was carried out at 30 days, 90 days, 150 days and 210 days. DE-mRNA results showed that 6,005 differentially expressed mRNA were found. 442, 2,304, 3,875, 2,642, 4,248 and 798 differentially expressed mRNAs were found in groups of 30 d vs 90d, 30 d vs 150d, 30 d vs 210d, 90 d vs 150d, 90 d vs 210d and 150 d vs 210 d (in which the control group is the one in front of vs). Upregulated and downregulated mRNAs in different groups were 298/144, 1425/879, 1932/1943, 1468/1174, 2005/2,243 and 373/425 respectively (Figure 3A and Supplementary Table S5-1).

DE-lncRNA results showed that there were 16, 156, 321, 158, 245 and 85 differentially expressed lncRNAs in 30 d vs 90d, 30 d vs 150d, 30 d vs 210d, 90 d vs 150d, 90 d vs 210d and 150 d vs 210 d (in which the control group was in front of vs). There were 10/6, 88/68, 260/61, 65/93, 162/83 and 62/23 lncRNAs upregulated and downregulated in each difference group, respectively (Figure 3B and Supplementary Table S5-3).

DE-miRNA results showed that there were 93, 115, 187, 141, 135 and 53 differentially expressed miRNAs in 30 d vs 90d, 30 d vs 150d, 30 d vs 210d, 90 d vs 150d, 90 d vs 210d and 150 d vs 210 d (in which the control group was in front of vs). Upregulated and downregulated miRNAs in different groups were 48/45, 32/83, 74/113, 47/94, 61/74 and 39/14, respectively (Figure 3C and Supplementary Table S5-2).

223, 218, 279, 254, 282 and 119 differentially expressed circRNAs were found in the different groups of 30 d vs 90d, 30 d vs 150d, 30 d vs 210d, 90 d vs 150d, 90 d vs 210d and 150 d vs 210d, respectively. There were 115/108, 139/79, 140/139, 168/86, 146/136 and 37/82 circRNAs upregulated and downregulated in each difference group (Figure 3D and Supplementary Table S5-4).

### 3.4 Functional enrichment analysis of differentially expressed mRNA

**FIGURE 4:**
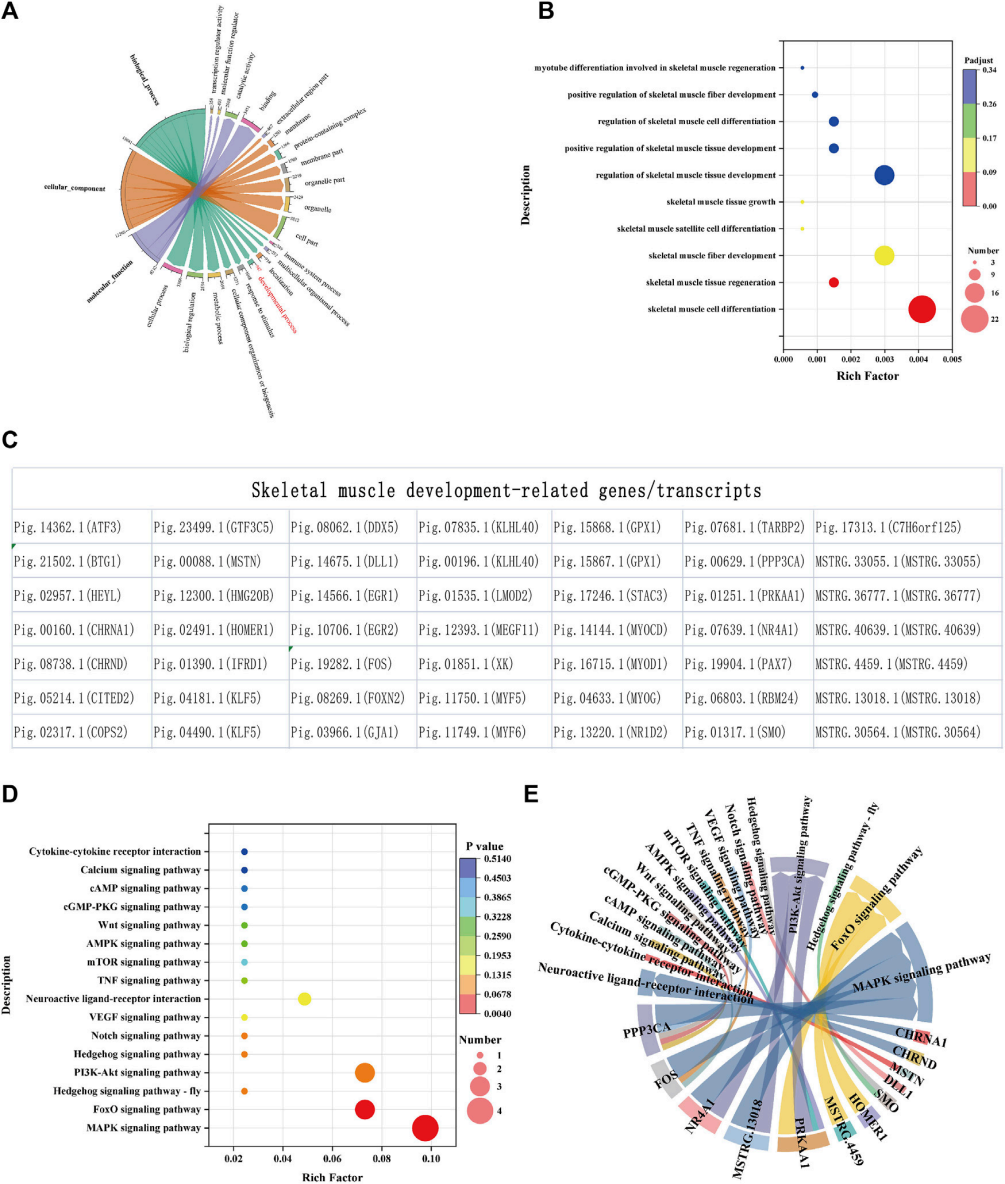
Functional annotation and enrichment analysis of differentially expressed mRNAs. **(A)** GO annotation of mRNA. GO enrichment **(B)**, transcripts **(C)** and KEGG **(D)** related to skeletal muscle development during development. **(E)** Signal pathways involved in key genes. The size and color of the bubble represent the number of genes enriched in the term or pathway and enrichment significance, respectively. The chord width indicates the number of mRNAs, and the chord arrows indicate the terms or pathways involved.

Among the 6,090 differentially expressed transcripts, 947 transcripts enriched GO terms in the developmental process (Figure 4A), of which 10 were enriched in terms related to skeletal muscle development (Figure 4B), mainly in GO:0035914, GO:0043403, GO:0048741 and other terms, corresponding to regulating skeletal muscle fiber development, skeletal muscle satellite cell differentiation, skeletal muscle tissue growth and regeneration (Supplementary Table S6-1). There are 49 transcripts of 10 terms related to skeletal muscle development, including 40 known genes and 6 novel genes (Figure 4C and Supplementary Table S6-1).

To clarify the signaling pathways involved in 46 genes related to skeletal muscle, KEGG analysis was carried out and the results showed that they were all enriched in signal transduction pathways such as MAPK, FoxO, Hedgehog, PI3K-Akt, Notch and VEGF (Figures 4D,E and Supplementary Table S6-2).

### 3.5 Analysis of skeletal muscle related proteins by PPI and transcription factors

**FIGURE 5:**
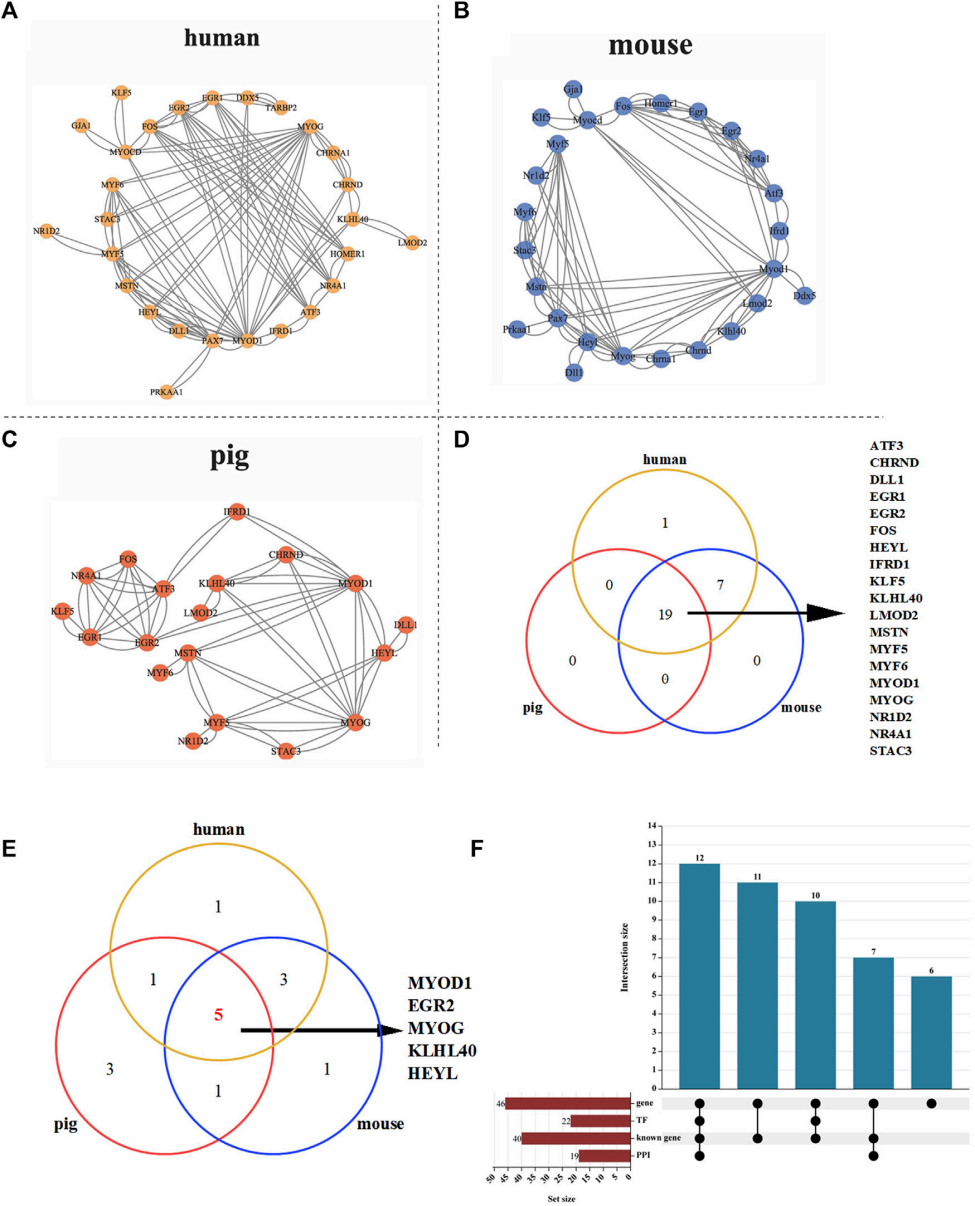
PPI analysis of 40 skeletal muscle related proteins. **(A)** PPI analysis of 39 proteins in human. **(B)** PPI analysis of 39 proteins in mouse. **(C)** PPI analysis of 39 proteins in pig. **(D)** Protein Venn analysis of human, mouse and pig. **(E)** Venn analysis of the top 10 core proteins in human, pig and mouse. **(F)** Upset analysis of genes, transcription factors and PPI.

To further analyze whether there is interaction between proteins encoded by 40 known genes related to skeletal muscle, we conducted protein interaction analysis in three different species: human, mouse, and pig. The results showed that there were 27, 26 and 19 proteins interacting with each other in human, mouse and pig, respectively (Figures 5A–C). Furthermore, Venn analysis was carried out on the interacting proteins in three species, and it was found that 19 proteins in pigs interacted stably, which indicated that the protein interaction relationship of these 19 proteins in pigs was stable (Figure 5D). Transcription factor analysis revealed that 12 of the 19 interacting proteins were transcription factors. To find the core regulatory proteins, centiscape 2.2 was used to calculate the betweenness of proteins interacting with each other in the three species. Venn analysis was performed on the top 10 proteins, and it was found that MYOD1, EGR2, MYOG, KLHL40 and HEYL occupy the core regulatory proteins in the three species (Figure 5E and Supplementary Table S7-(13).

Transcription factor analysis revealed that 22 of 40 proteins were transcription factors and 12 of 19 interacting proteins were transcription factors (Figure 5F). Seven transcripts (Pig.14566.1, Pig.10706.1, Pig.11750.1, Pig.04181.1, Pig.04490.1, Pig.11750.1, Pig.13220.1) showed the lowest expression levels at 90 days after birth. Then it rises gradually, reaching the highest level at 210 days after birth (Supplementary Figure S3 and Supplementary Table S8). These proteins may regulate skeletal muscle development by affecting gene transcription.

### 3.6 Construction of ceRNA interaction regulation network

**FIGURE 6:**
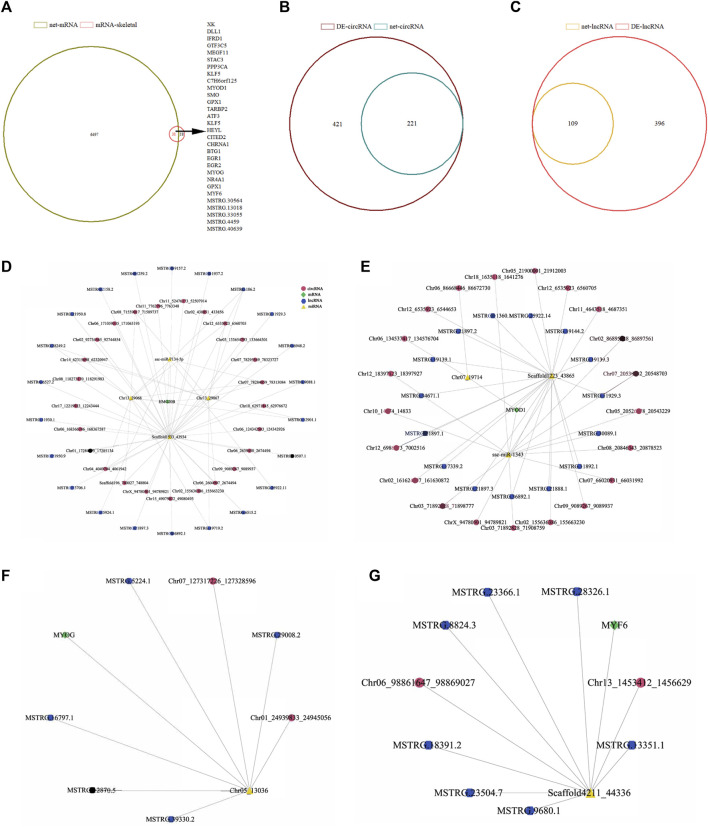
CircRNA/lncRNA-miRNA-mRNA interaction network analysis of differentially expressed genes related to skeletal muscle of Ningxiang pigs. **(A)** Venn analysis results of 49 differentially expressed genes associated with skeletal muscle and mRNA in ceRNA network. **(B, C)** Venn analysis results of differentially expressed circRNAs and lncRNAs in ceRNA networks, **(D–G)** Interaction network of skeletal muscle development marker genes (*HMG20B*, *MYOD1*, *MYOG*, *MYF6*). The pink and blue dots represent circRNA and lncRNA, respectively. Yellow triangles represent miRNA and green diamonds represent mRNA.

The competitive endogenous RNA (ceRNA) network constructed by miRanda prediction and expression correlation includes 824 miRNAs, 1,664 circRNAs, 608 lncRNAs and 6,528 mRNAs, with 9,624 nodes and 28,275 ceRNA pairs (Supplementary Table S9-1). To construct a skeletal muscle-related ceRNA network, 49 differentially expressed transcripts related to muscle development and mRNAs in ceRNA network were analyzed by venn and it was found that 31 of them were in the ceRNA network (Figure 6A). Further analysis showed that 31 skeletal muscle-related genes combined with 59 miRNAs and 59 miRNAs combined with 224 lncRNAs and 677 circRNAs, respectively. The ceRNA regulatory network related to skeletal muscle development includes 991 nodes and 1562 relationship pairs. There are 1,100 circRNA-miRNA pairs, 376 lncRNA-miRNA pairs and 86 mRNA-miRNA pairs in the network (Supplementary Figure S4).

To test the authenticity of ceRNA regulatory network, we confirmed it by the positive correlation between ceRNA and ceRNA. Venn analysis was carried out on the lncRNA and circRNA combined with miRNA in the network and the differentially expressed lncRNAs and circRNAs. There were 221 and 109 differentially expressed RNAs in the network (Figures 6B,C). The reconstructed ceRNA interactive network contains 420 nodes, 412 circRNA-miRNA pairs, 226 lncRNA-miRNA pairs and 86 mRNA-miRNA pairs (Supplementary Figure S5 and Supplementary Table S9-2). *HMG20B*, *MYOD1*, *MYOG* and *MYF6* are marker genes related to muscle development and these four genes were selected to construct the ceRNA regulatory network of skeletal muscle development in Ningxiang pigs. The ceRNA network of *HMG20B* contains 4 miRNAs, 22 lncRNAs and 25 circRNAs (Figure 6D). In the network, *MYOD1* is regulated by 3 miRNAs, 15 lncRNAs and 21 circRNAs (Figure 6E). *MYOG* is regulated by 2 miRNAs, 5 lncRNAs and 2 circRNAs (Figure 6F). The regulation of *MYF6* is performed by 1 miRNA, 7 lncRNAs and 2 circRNAs (Figure 6G).

### 3.7 Verification of RNA-Seq differential expression results by RT-qPCR

To further verify the sequencing results of whole transcriptome, 4 DE-mRNAs (*HMG20B, MYF6, MYOD1, MYOG*), 9 DE-lncRNAs (MSTRG.21897.3, MSTRG.21897.3, MSTRG.6515.2, MSTRG.6515.2, MSTRG.18391.2, MSTRG.23366.1, MSTRG.11929.3, MSTRG.30089.1, MSTRG.16797.1) and 6 DE-circRNAs (Chr06_98861647_98869027、Chr13_1453412_1456629, Chr02_161624907_161630872, Chr09_9089267_9089937, Chr07_127317226_127328596) were randomly selected to detect their expression patterns by RT-qCPR. As shown in Supplementary Figures S6A-C, RT-qPCR results imply that the expression patterns of the selected RNA are highly consistent in the two methods.

**FIGURE 7:**
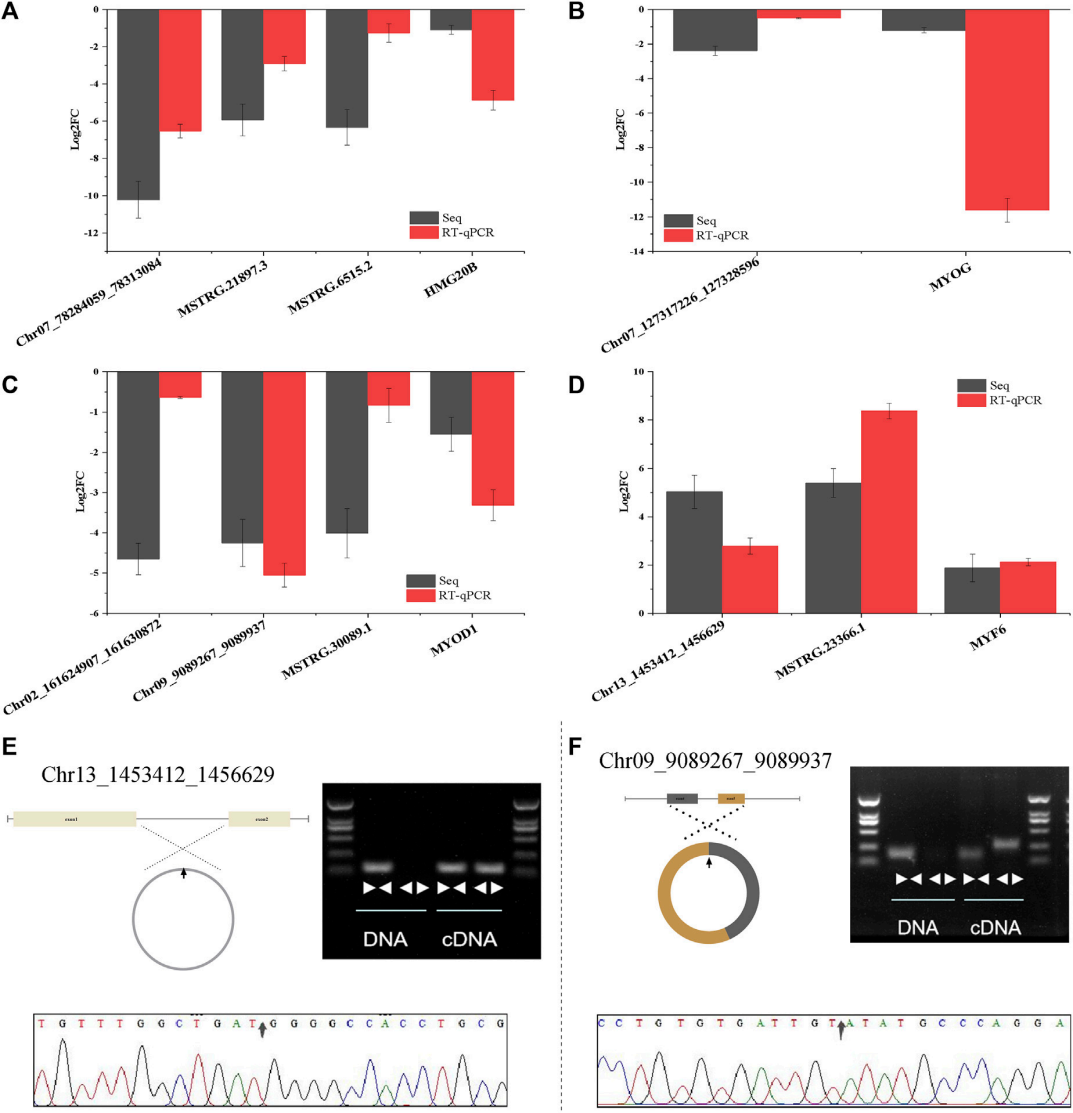
CeRNA interaction network and circRNA cyclization validation. **(A–D)** Verification of ceRNA network of four skeletal muscle related genes (*HMG20B, MYOG, MYOD1, MYF6*) by RT-qPCR. **(E–F)** Cyclization validation of Chr13_1453412_1456629 and Chr09_90892672_9089937. “face to face” and “back to back” are convergent primer and divergent primer amplification respectively. The arrow in the sanger sequencing peak diagram is back-splicing junction site. Data are presented as the mean ± SEM. n = 3.

To verify the ceRNA network, the ceRNA pairs in *HMG20B*, *MYOD1*, *MYOG* and *MYF6* were selected, and the sequencing results were verified by RT-qPCR. The results showed that the expression levels of each ceRNA pair were consistent (Figures 7A–D). The PCR amplification products of Chr13_1453412_1456629 and Chr09_9089267_9089937 were verified by agarose gel electrophoresis. Divergent primers were not amplified in gDNA, but in cDNA and convergent primers were amplified in both DNA. The products amplified by divergent primers were conducted by Sanger sequencing to determine the back-splicing junctions (Figures 7E,F). These results suggest that the results of whole transcriptome sequencing and bioinformatics analysis pipeline in this study have a high reliability.

## 4 Discussion

Whole transcriptome sequencing is a key technology to identify mRNA and ncRNA and is widely used to elucidate molecular mechanisms of growth and development, diseases and other related fields (O'Donohue et al., 2023). In this study, 20,630 mRNAs, 29,963 circRNAs, 2,683 lncRNAs and 986 miRNAs were identified in the *longissimus dorsi* muscle of Ningxiang pig at four stages (30, 90, 150, 210 days after birth) after mapping the whole transcribed sequence with the genome of Ningxiang pig. Hong et al. sequenced the transcriptome of the *longissimus dorsi* of Duroc pigs at three embryonic ages (33, 65 and 90 days of gestation) and mapped it with the Duroc genome and identified 84,693 circRNAs (Hong et al., 2019). Li et al. sequenced the biceps femoris and soleus muscles of Duroc×meishan binary hybrid pigs and identified 16,342 circRNAs by comparing the genomes of Duroc pigs (Li et al., 2020). These studies have shown that more circRNAs can be identified by mapping the sequence of transcriptome with the genome of the same breed of pig, because the reference genome information is more accurate.

The differential expression of intracellular molecules is a vital signal of function. In this study, 6,090 differentially expressed genes were identified by whole transcriptome sequencing. Through GO enrichment analysis, 46 differentially expressed genes were significantly enriched in skeletal muscle related items, including 40 known genes such as *MSTN*, *MYF5*, *MYOG*, *PAX7, HEYL*, *etc.*, and 6 novel genes (*MSTRG.30564, MSTRG.36777, MSTRG.40639, MSTRG.13018, MSTRG.33055, MSTRG.4459*), *PAX7, MYOD*, *MYOG*, *MYF5*, *MYHC*, *MSTN*, *MRF* and other genes have been confirmed to be involved in the regulation of skeletal muscle development were also found in this paper (Wang et al., 2022c; Shirakawa et al., 2022; Vicente-García, Hernández-Camacho, and Carvajal, 2022). Some studies have found that *LMOD2* is involved in the regulation of actin filaments in muscle (Conley et al., 2001). Cong et al. reported that *STAC3* has an important effect on muscle fiber hypertrophy and muscle fiber composition of skeletal muscle in postnatal mice (Cong et al., 2016). Zhao et al. reported that the absence of *MSTN* can promote the differentiation of bovine myoblasts (Zhao et al., 2022). These genes involved in skeletal muscle regulation reported above were also identified in the current study, which implies that the results of our study are highly reliable.

Gene transcription is a critical part of molecular function is regulated by transcription factors. In this study, transcription factor analysis results showed that 22 of the 40 genes, including *ATF3*, *NR4A3*, *FOS*, *EGR1* and *BTG1*, were transcription factors. Fukuda et al. found that *Hyel* can promote the proliferation of muscle stem cells in muscle atrophy (Fukuda et al., 2019). Mey et al. reported that *NR4A3* affects skeletal muscle hypertrophy by regulating MSTN expression in skeletal muscle (Mey et al., 2019). *FOS* can participate in the differentiation process of muscle cells (Almada et al., 2021) and the formation of myotubes will be inhibited when *FOS* expression is reduced (Zhao Q. et al., 2015). Mohtar et al. found that *EGR1* could regulate tendon formation (Mohtar et al., 2019). Studies have revealed that *BTG1* can inhibit myoblast proliferation and stimulate myoblast differentiation (Bakker et al., 2004; Busson et al., 2005). Transcription factors initiate transcription mainly by binding directly or indirectly to the promoter region of the target gene. To further explore whether there is an interaction among these transcription factors, PPI analysis was performed on 40 genes in human, pig and mouse. There were significant interactions among the 19 proteins by PPI analysis. 12 of the 19 interacting proteins are transcription factors (ATF3, EGR1, EGR2, FOS, HEYL, KLF5, MYF5, MYF6, MYOD1, MYOG, NR1D2, NR4A1). Nandagopal et al. found that *DLL1* upregulated the expression of HEYL gene by activating Notch signaling pathway to promote myogenesis (Nandagopal et al., 2018). Jeong et al. found that ATF3 acts as a cofactor of FOS to activate bone cell differentiation (Jeong et al., 2017), and whether this interaction is involved in skeletal muscle development needs further verification. Whether these new genes (*MSTRG.30564, MSTRG.36777, MSTRG.40639, MSTRG.13018, MSTRG.33055, MSTRG.4459*) are involved in the regulation of skeletal muscle development needs further experimental verification.

Genes function not only in cells, but also through intercellular communication to achieve signal transduction and amplification, thereby producing biological effects. In this study, KEGG pathway enrichment analysis showed that these genes (*PPP3CA, FOS, NR4A1, MSTRG.13018, PRKAA1, MSTRG.4459, HOMER1, SMO, DLL1, MSTN, CHRND, CHRNA1*) mainly regulate the development of skeletal muscle through signal transduction pathways such as MAPK, FoxO, mTOR, PI3K-Akt, Notch and VEGF. Leger et al. found that FoxO/Akt signal pathway regulates skeletal muscle hyperplasia and atrophy (Leger et al., 2006). Studies have found that FoxO/PI3K-Akt signals are the key pathways that affect muscle fiber transformation (Cao et al., 2022). Han et al. found that mTOR signaling pathway is involved in regulating skeletal muscle autophagy and other diseases (Han et al., 2022). Zhao et al. found that PI3K-Akt signaling pathway regulates the differentiation of bovine myoblasts (Zhao et al., 2022). These signal pathways are related to skeletal muscle development, which is consistent with the results of this study.

Some studies have shown that circRNA can act as a molecular sponge of miRNA to regulate the development of animal tissues by relieving the inhibition of miRNA on target genes and circRNA presents a time-specific expression pattern in tissues (Memczak et al., 2013b; Shi et al., 2021). In this study, the *longissimus dorsi* muscle of Ningxiang pigs was sequenced at four postnatal periods (30, 90, 150 and 210 days after birth). Through bioinformatics analysis, the circRNA/lncRNA-miRNA-mRNA regulatory network was constructed, which was centered on *HMG20B*, *MYOG*, *MYF6* and *MYOD1*, involved in the regulation of skeletal muscle fiber type transformation after birth. Sun et al. reported the ceRNA network of miR-21-3p/CDK16 mediated by circCSDE1, which regulates pig muscle development (Sun et al., 2022). Li et al. constructed the regulatory network of porcine skeletal muscle differentiation with circIGF1R/miR-16/mRNA through sequencing and bioinformatics analysis (Li et al., 2023). It is reported that circRNA can participate in the regulation of muscle development by affecting the expression of marker genes in skeletal muscle. For example, Chen et al. studied that circ*MYBPC1* could promote the differentiation of bovine myoblasts and alleviate the inhibition of the target gene *MYHC* through competitive binding of miR-23a (Chen et al., 2021). In this study, ceRNA regulatory networks of four marker genes, *MYOG, MYOG, MYF6* and *MYOD1*, were constructed, among which the ceRNA regulatory network of HMG20B was also reported in Duroc pigs (Hong et al., 2019). Hong et al. sequenced Duroc *longissimus dors* muscle at three embryonic stages (33, 65, 90 days of pregnancy) and constructed the ceRNA regulatory network for skeletal muscle development (Hong et al., 2019). Li et al. constructed a circRNA-miRNA-mRNA regulatory network affecting the transformation of skeletal muscle fibers in pigs by sequencing the biceps femoris and soleus muscle of Duroc-Mashen dual hybrid pigs (Li et al., 2020). Wang et al. compared circRNA differentially expressed in the *longissimus dorsi* muscle of western lean meat breeds and local Chinese breed Huainan pigs and constructed a network regulating ceRNA for meat quality traits (Wang et al., 2019). Previous studies on muscle development mainly focused on high-throughput sequencing of different embryonic stages or different types of muscle tissue, but the transformation of muscle fiber type mainly occurred after birth. The *HMG20B* regulating the development of skeletal muscle fibers found in this study was consistent with the ceRNA results found by Hong et al. during the embryonic period (Hong et al., 2019). The ceRNA interaction network that regulates skeletal muscle marker genes *MYOD1*, *MYF6* and *MYOG* has not been reported, and further studies are needed to verify the authenticity of this network through a series of experiments *in vivo* and *in vitro*.

## 5 Conclusion

In conclusion, through comparative analysis of muscle histological parameters of *longissimus dorsi* muscle of Ningxiang pigs at different growth stages, it was found that the diameter of muscle fiber reached the maximum at 150 days. The slow muscle fiber transformation showed a pattern of initial decrease followed by an increase. The profiles of mRNA and ncRNA during skeletal muscle fiber type transformation were analyzed by whole transcriptomic sequencing technology, and 40 key genes including *MSTN, MYF6, MYOG, PAX7, MYOD1, HEYL* and 6 new genes involved in skeletal muscle fiber development were identified. The ceRNA regulatory network of cricRNA/lncRNA-miRNA-mRNA with skeletal muscle development-related genes as the core was constructed. It provides a new insight into genetic improvement of pork quality, skeletal muscle development and molecular mechanism of muscle related diseases.

## Data Availability

The datasets presented in this study can be found in online repositories. The names of the repository/repositories and accession number(s) can be found below: https://www.ncbi.nlm.nih.gov/bioproject/, PRJNA721288.
