# Supplementary material for: Integrated analysis of circRNA, lncRNA, miRNA and mRNA to reveal the ceRNA regulatory network of postnatal skeletal muscle development in Ningxiang pig
Source: Front Cell Dev Biol. 2023 Jul 3;11:1185823. doi: 10.3389/fcell.2023.1185823 (PMC10350537; doi:10.3389/fcell.2023.1185823)
Supplement: Supplementary file 1 [file DataSheet1.ZIP › Supplementary_Material .docx]

Supplementary Material

Integrated analysis of CircRNA, lncRNA, miRNA and mRNA to reveal the ceRNA regulatory network of postnatal skeletal muscle development in Ninxiang pig

Zonggang Yu^1^, Xueli Xu^1^, Nini Ai^1^, Kaiming Wang^1^, Peiwen Zhang^1^, Xintong Li^1^, Fusui Liu^1^, Xiaolin Liu^1^, Jun Jiang^1^, Jingjing Gu^1^, Ning Gao^1^, Haiming Ma^1, 2*^

*** Correspondence:** Haiming Ma, mahaiming2000@163.com

# Supplementary Figures and Tables

## Supplementary Figures

For supplementary figures, please check the PDF in the supplementary materials.

**Supplementary Figure S1.** Gene region distribution (A) and chromosome (B) distribution of clean reads.

**Supplementary Figure S2.** Cluster analysis of RNA at different four stages (30d, 90d, 150d and 210d after birth). (A-D) Cluster analysis of miRNA, lncRNA, circRNA and mRNA.

**Supplementary Figure S3.** Temporal expression profiles of transcripts of 12 skeletal muscle-related transcription factors. X axis is at different stages after birth (30d, 90d, 150d and 210d), and Y axis is the TPM of expression. Data are presented as the mean ± STD. n = 3.

**Supplementary Figure S4.** Overview of ceRNA (circRNA/lncRNA-miRNA-mRNA) regulatory networks of skeletal muscle related genes.

**Supplementary Figure S5.** circRNA and lncRNA were screened by differential expression to construct a ceRNA (circRNA/lncRNA-miRNA-mRNA) regulatory network for regulating skeletal muscle-related genes.

**Supplementary Figure S6.** RT-qPCR verification of differentially expressed RNA. (A-C) the differentially expressed lncRNA, mRNA and circRNA were verified by RT-qPCR.

## Supplementary Tables

Supplementary table, please check the xlsx file in the supplementary materials.

**Supplementary Table S1.** Primer sequence used for RT-qPCR verification.

**Supplementary Table S2.** Details of raw reads and clean reads of long RNA and small RNA. (Table S2-1) Raw reads and quality control parameters Details of long RNA. (Table S2-2) Raw reads and quality control parameters details of small RNA.

**Supplementary Table S3.** Detailed of Reads chromosome and gene distribution. (Table S3-1) The distribution of reads on chromosomes. (Table S3-2) The distribution of reads in the gene region.

**Supplementary Table S4.**  Screening of expression levels for four types of RNA. (Table S4-1) mRNA screened by TPM>1. (Table S4-2) miRNA screened by TPM>0.05. (Table S4-3) LncRNA screened by TPM>0.05. (Table S4-4) CircNRNA screened by RPM>0.05.

**Supplementary Table S5.** Details of differential expression of four types of RNA. (Table S5-1) Table of differentially expressed mNRA details. (Table S5-2) Table of differentially expressed miNRA details. (Table S5-3) Table of differentially expressed lncNRA details. (Table S5-4) Table of differentially expressed circNRA details.

**Supplementary Table S6.** Functional enrichment Analysis of mRNA. (Table S6-1) GO enrichment analysis of transcripts with TMP > 1 expression. (Table S6-2) KEGG enrichment analysis of skeletal muscle related genes.

**Supplementary Table S7.** Betweenness analysis of protein-protein interaction among three spices. (Table S7-1) Centiscape2.2 analysis of human protein-protein interaction. (Table S7-2) Centiscape2.2 analysis of mouse protein-protein interaction. (Table S7-3) Centiscape2.2 analysis of pig protein-protein interaction.

**Supplementary Table S8.** Temporal expression profiles of transcripts of 12 skeletal muscle-related transcription factors

**Supplementary Table S9.** CeRNA regulatory networks of predicted by miRanda and skeletal muscle related genes. (Table S9-1) CeRNA regulatory network predicted by miRanda software. (Table S9-2) CeRNA regulatory network related to skeletal muscle.
