# Supplementary figures and images for: Integrated analysis of circRNA, lncRNA, miRNA and mRNA to reveal the ceRNA regulatory network of postnatal skeletal muscle development in Ningxiang pig
Source: Front Cell Dev Biol. 2023 Jul 3;11:1185823. doi: 10.3389/fcell.2023.1185823 (PMC10350537; doi:10.3389/fcell.2023.1185823)

A

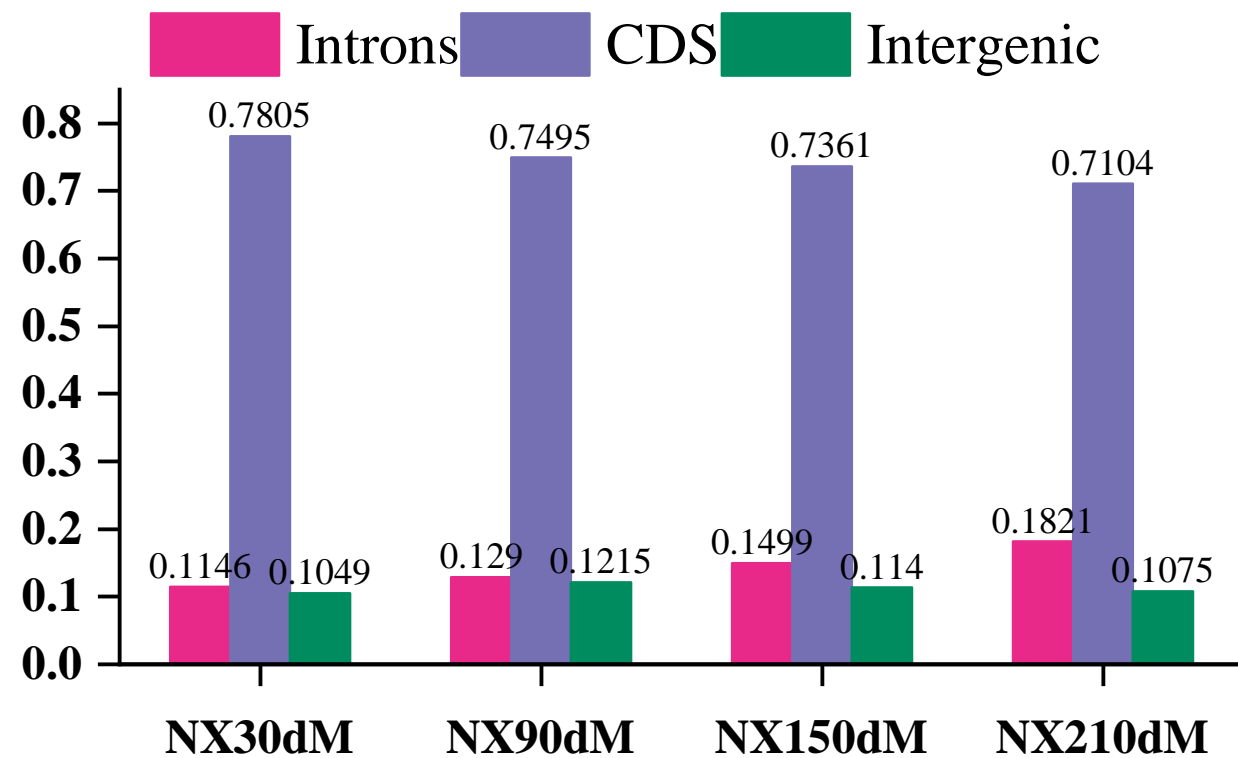

B

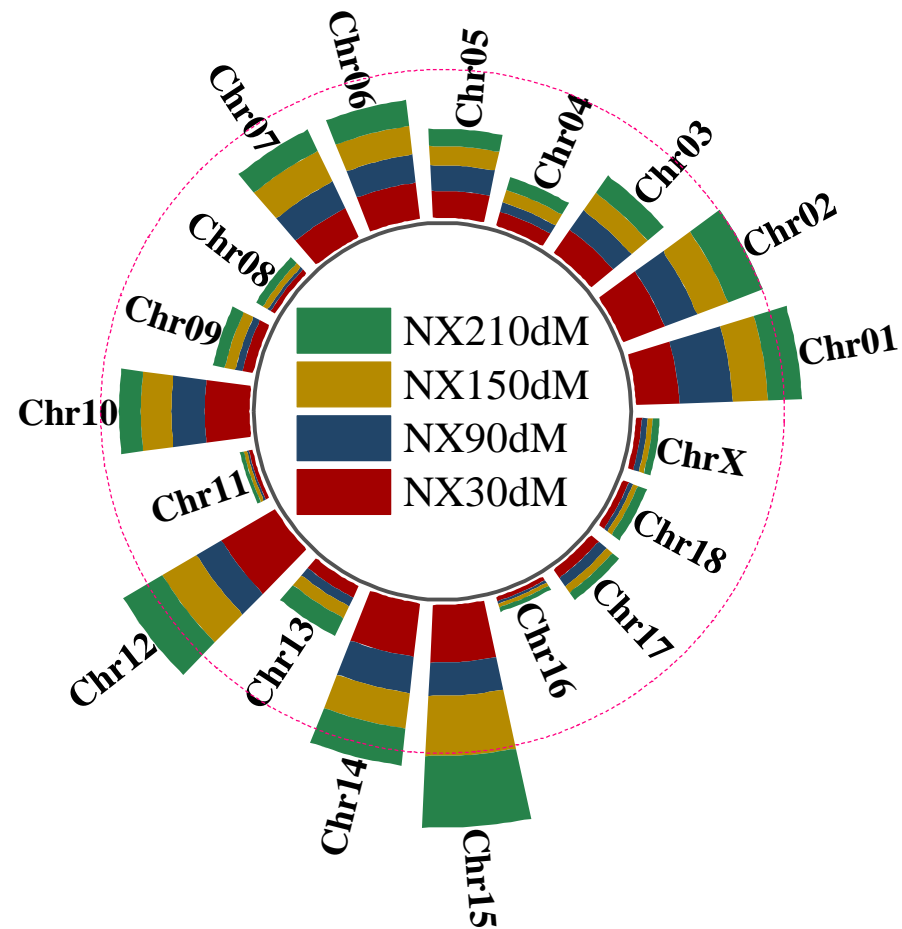

Supplement: Supplementary file 1 [file DataSheet1.ZIP › Supplementary Figure S1.pdf]

A

miRNA

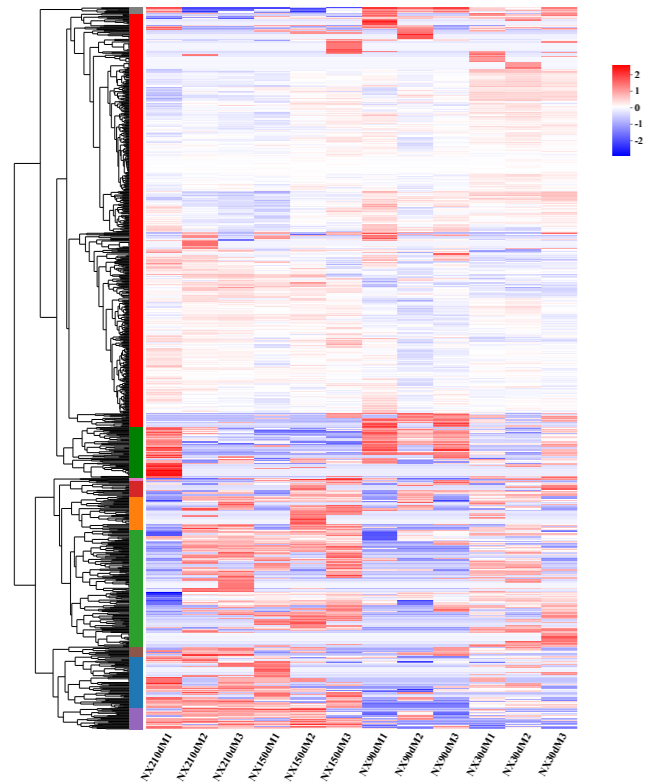

B

lncRNA

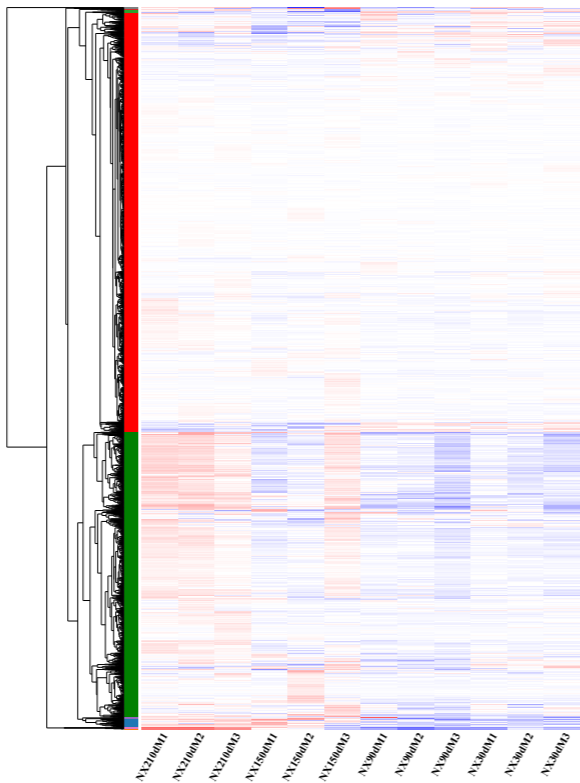

C

circRNA

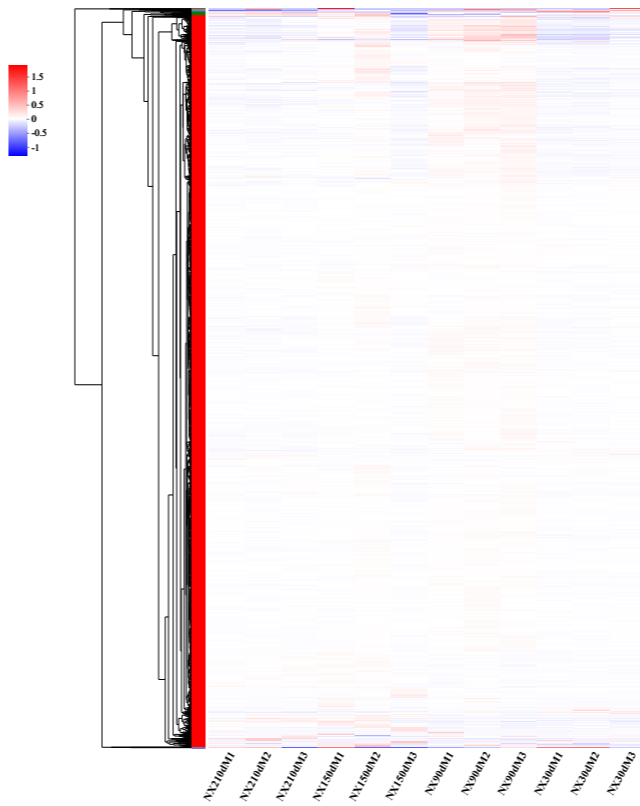

D

mRNA

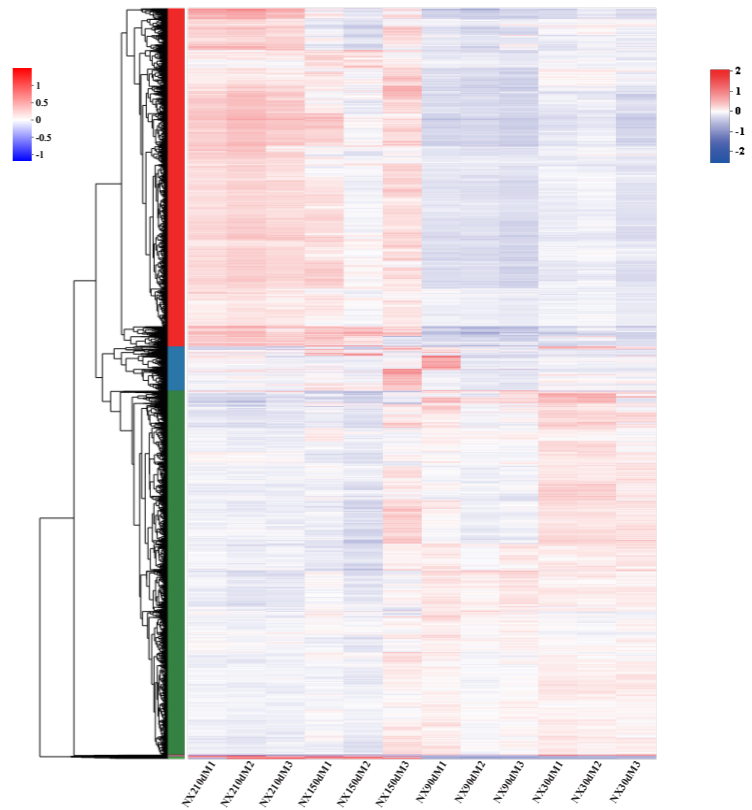

Supplement: Supplementary file 1 [file DataSheet1.ZIP › Supplementary Figure S2.pdf]

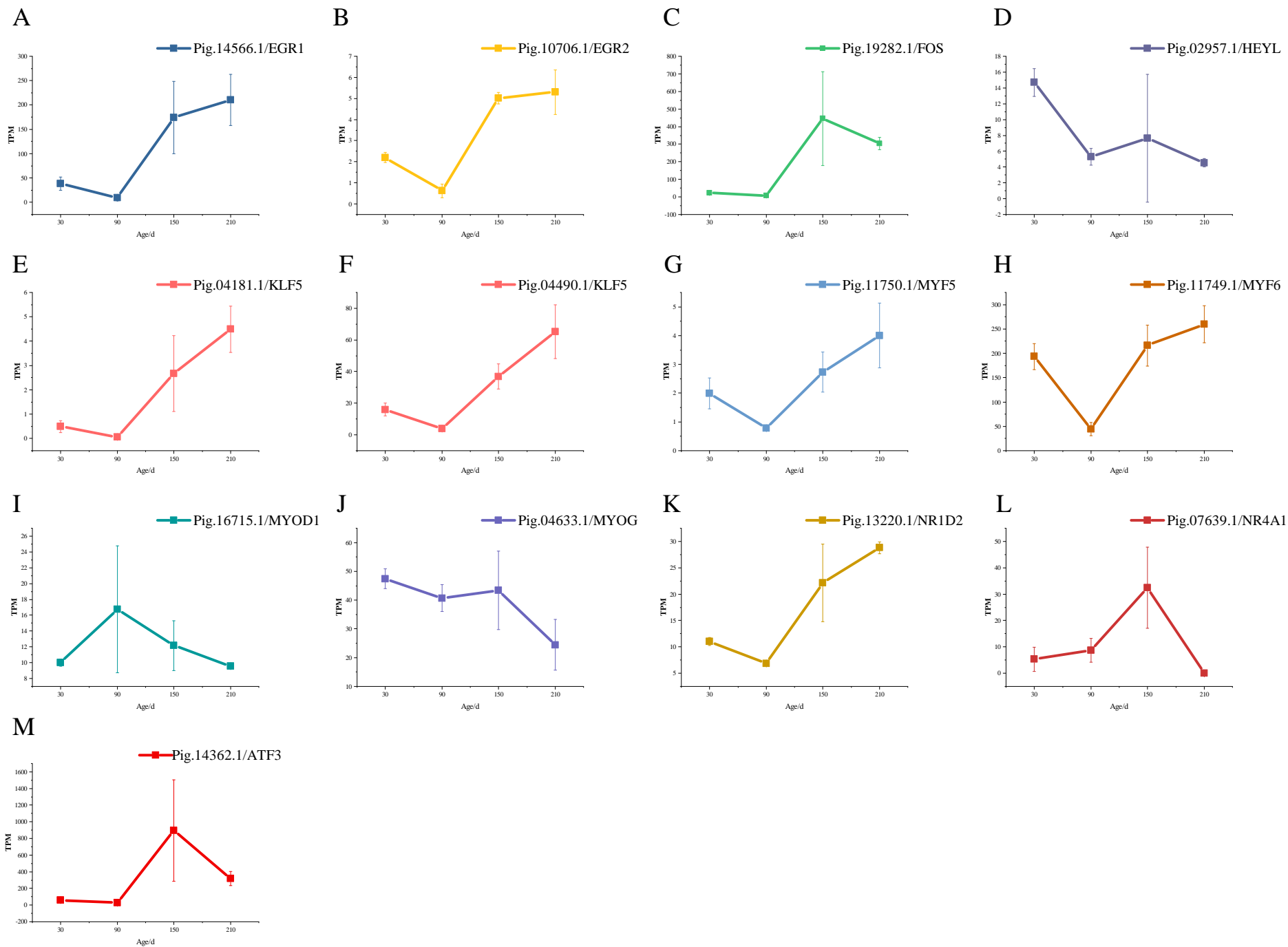

Supplement: Supplementary file 1 [file DataSheet1.ZIP › Supplementary Figure S3.pdf]

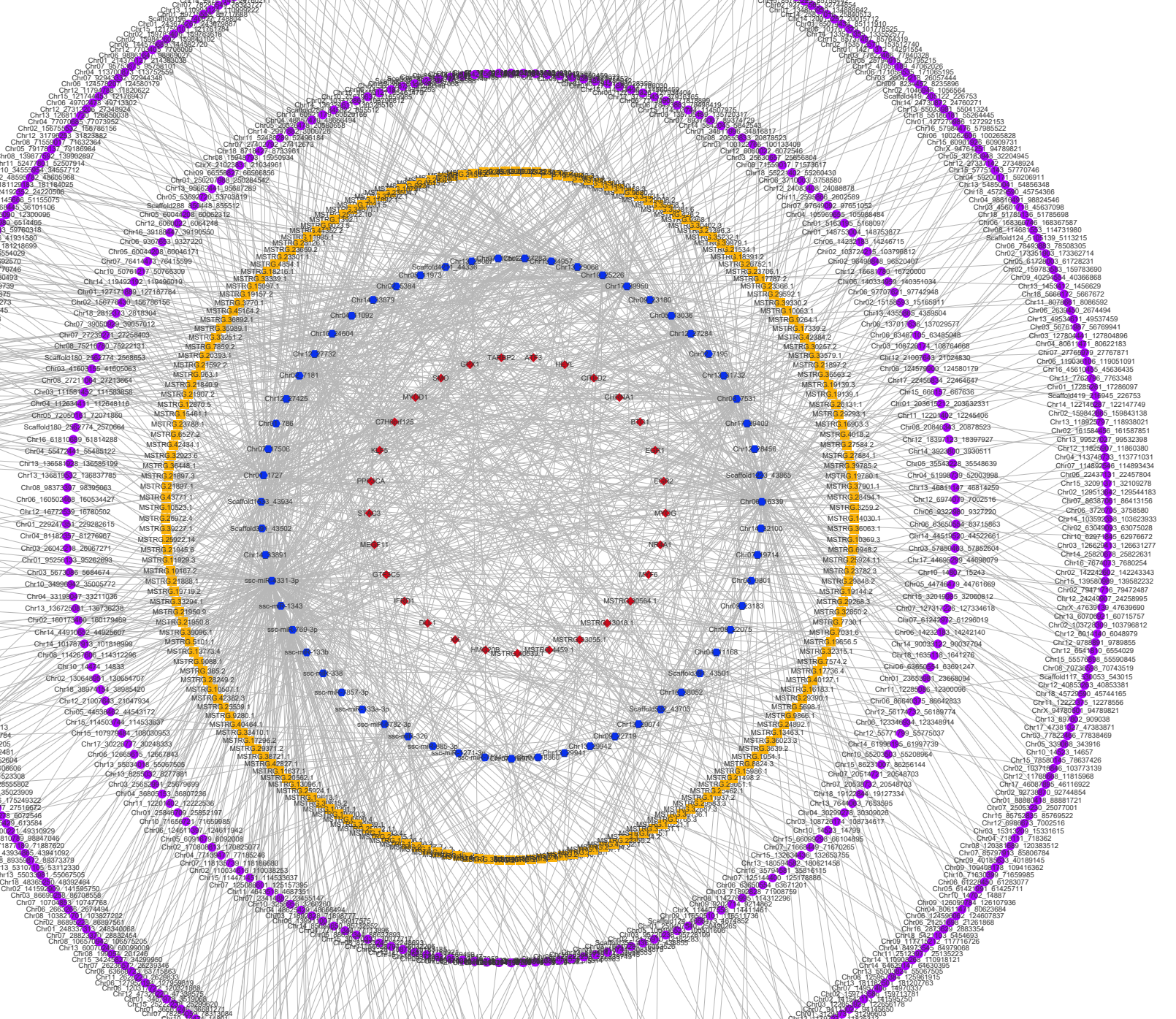

Supplement: Supplementary file 1 [file DataSheet1.ZIP › Supplementary Figure S4 .pdf]

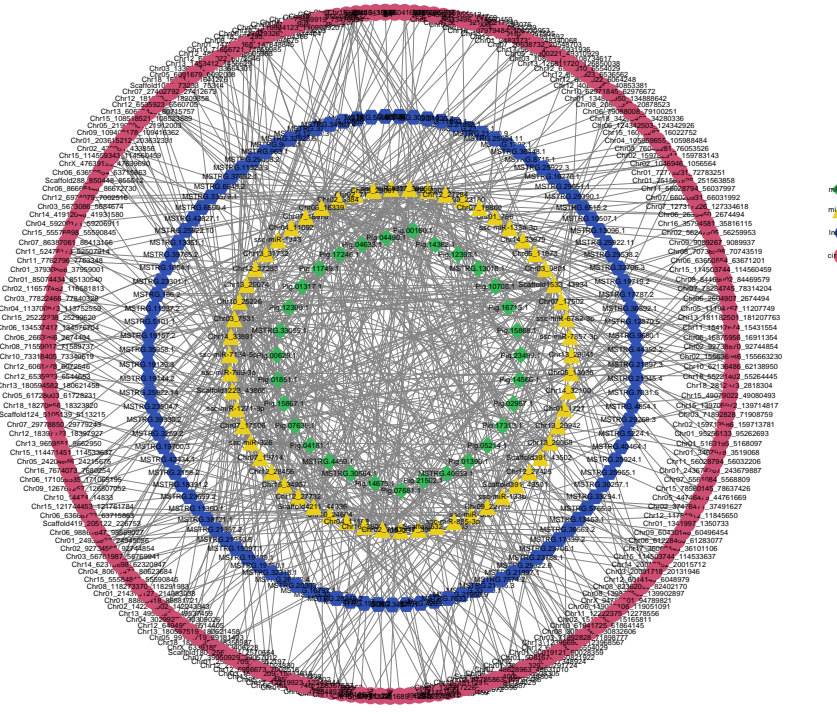

Supplement: Supplementary file 1 [file DataSheet1.ZIP › Supplementary Figure S5 .pdf]

A

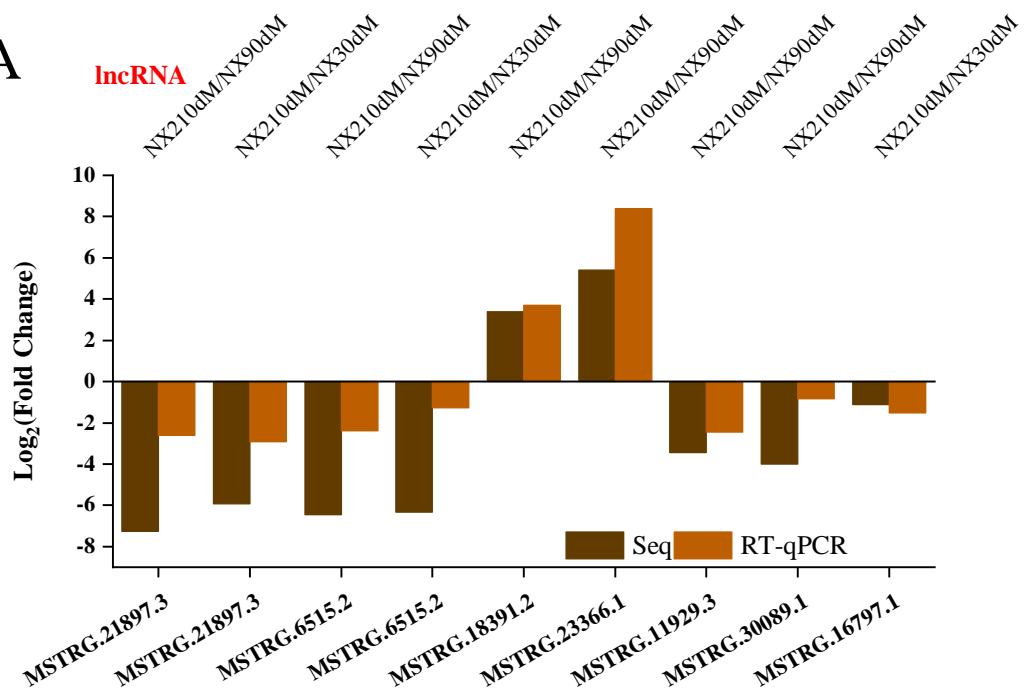

B

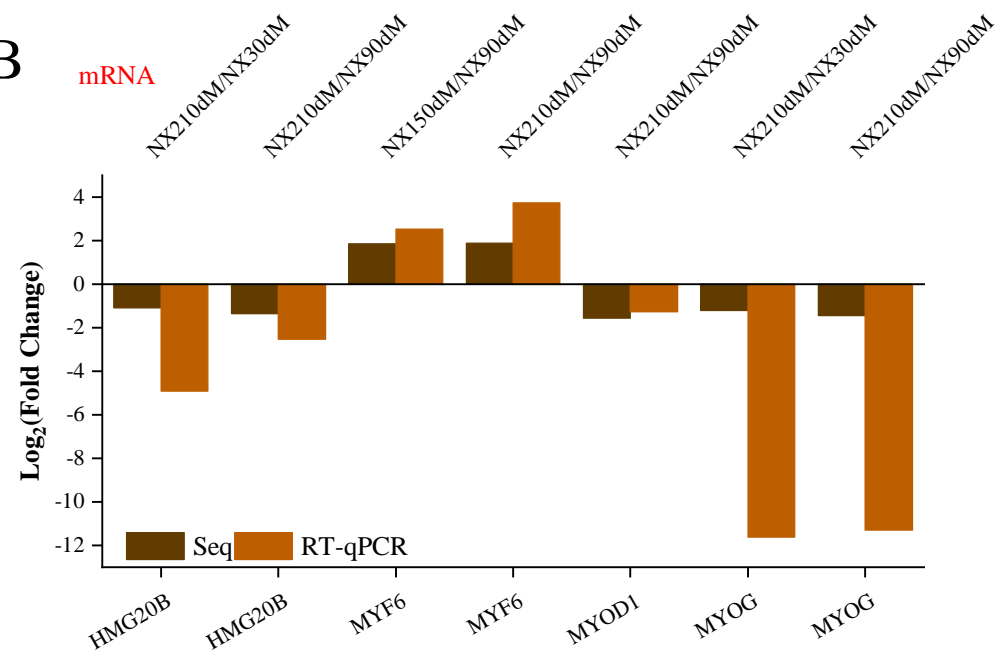

C

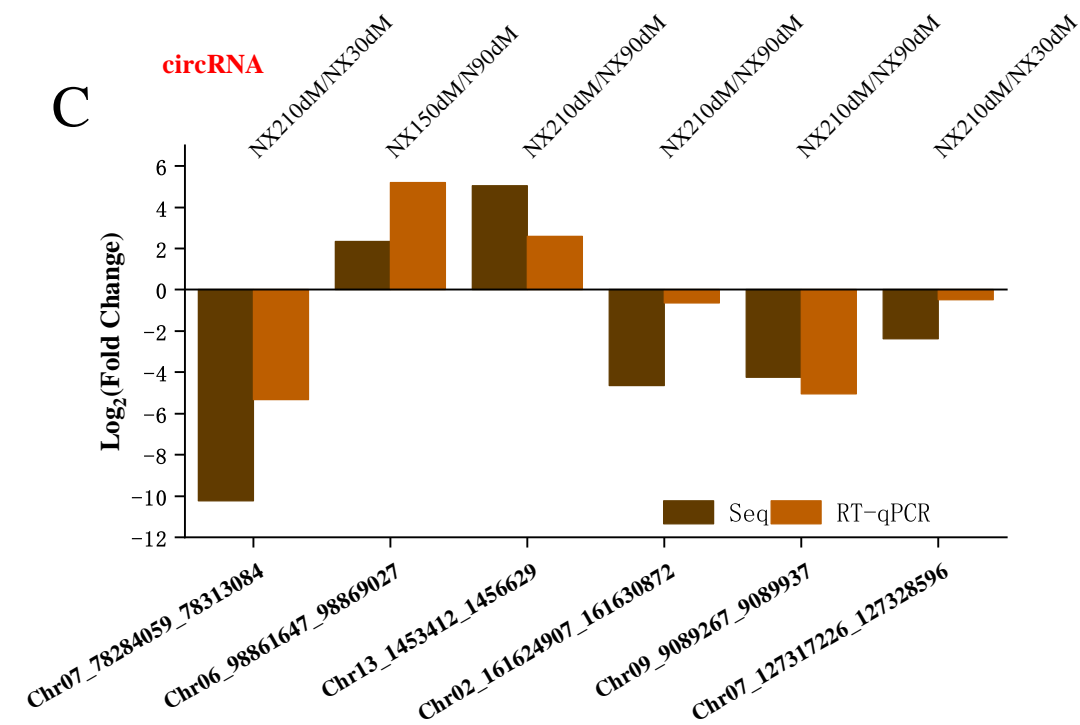

Supplement: Supplementary file 1 [file DataSheet1.ZIP › Supplementary Figure S6.pdf]
